# The Methylcitrate Cycle and Its Crosstalk with the Glyoxylate Cycle and Tricarboxylic Acid Cycle in Pathogenic Fungi

**DOI:** 10.3390/molecules28186667

**Published:** 2023-09-17

**Authors:** Zhicheng Huang, Qing Wang, Irshad Ali Khan, Yan Li, Jing Wang, Jiaoyu Wang, Xiaohong Liu, Fucheng Lin, Jianping Lu

**Affiliations:** 1 State Key Laboratory for Managing Biotic and Chemical Threats to the Quality and Safety of Agro-Products, College of Life Sciences, Zhejiang University, Hangzhou 310058, China; huangzhicheng1210@163.com (Z.H.); m15760206862_1@163.com (Q.W.); leeysmailbox@163.com (Y.L.); 2 Department of Agriculture, The University of Swabi, Khyber 29380, Pakistan; irshad_1242000@yahoo.com; 3 State Key Laboratory for Managing Biotic and Chemical Threats to the Quality and Safety of Agro-Products, Institute of Plant Protection and Microbiology, Zhejiang Academy of Agricultural Sciences, Hangzhou 310021, China; wj9311@163.com (J.W.); wangjiaoyu78@sina.com (J.W.); fuchenglin@zju.edu.cn (F.L.); 4 Institute of Biotechnology, Zhejiang University, Hangzhou 310058, China; xhliu@zju.edu.cn

**Keywords:** acetyl-CoA, citrate synthase, growth, isocitrate lyase, 2-methylcitrate synthase, 2-methylisocitrate lyase, propionyl-CoA, virulence

## Abstract

In fungi, the methylcitrate cycle converts cytotoxic propionyl-coenzyme A (CoA) to pyruvate, which enters gluconeogenesis. The glyoxylate cycle converts acetyl-CoA to succinate, which enters gluconeogenesis. The tricarboxylic acid cycle is a central carbon metabolic pathway that connects the methylcitrate cycle, the glyoxylate cycle, and other metabolisms for lipids, carbohydrates, and amino acids. Fungal citrate synthase and 2-methylcitrate synthase as well as isocitrate lyase and 2-methylisocitrate lyase, each evolved from a common ancestral protein. Impairment of the methylcitrate cycle leads to the accumulation of toxic intermediates such as propionyl-CoA, 2-methylcitrate, and 2-methylisocitrate in fungal cells, which in turn inhibits the activity of many enzymes such as dehydrogenases and remodels cellular carbon metabolic processes. The methylcitrate cycle and the glyoxylate cycle synergistically regulate carbon source utilization as well as fungal growth, development, and pathogenic process in pathogenic fungi.

## 1. Introduction

Propionyl-CoA is an intermediate metabolite produced by organisms during metabolism, which is toxic to cells [1]. Propionate, amino acids (isoleucine, methionine, threonine, and valine), thymine, and odd chain fatty acids are catabolized to yield propionyl-CoA [2,3]. Propionate is the second most abundant organic acid naturally occurring in soil. Propionate inhibits the growth of microorganisms and is used as a common food preservative [4]. Four amino acids (isoleucine, methionine, threonine, and valine) account for about 15% of amino acid abundance in proteins of various environmental microorganisms [5]. Propionyl-CoA is also produced by cholesterol via side chain oxidation. After propionyl-CoA is produced, organisms have three pathways to catabolize propionyl-CoA. In animals and some bacteria, the methylmalonyl-CoA pathway is a pathway that metabolizes propionyl-CoA [3]. Propionyl-CoA is sequentially converted to methylmalonyl-CoA, succinyl-CoA, and malate, which is then metabolized to acetyl-CoA and glyoxylate [6]. Another propionyl-CoA metabolic pathway is the methylcitrate cycle present in fungi and some bacteria [7,8]. In the pathogenic fungus *Candida albicans*, Otzen et al. proposed a third propionyl-CoA metabolic pathway that propionyl-CoA is metabolized via a modified β-oxidation pathway [9]. In this β-oxidation pathway, propionyl-CoA is sequentially converted to acrylyl-CoA, 3-hydroxypropionyl-CoA, 3-hydroxypropionate, and malonate semialdehyde, which is then metabolized to acetyl-CoA or acetate [9].

**Figure 1:**
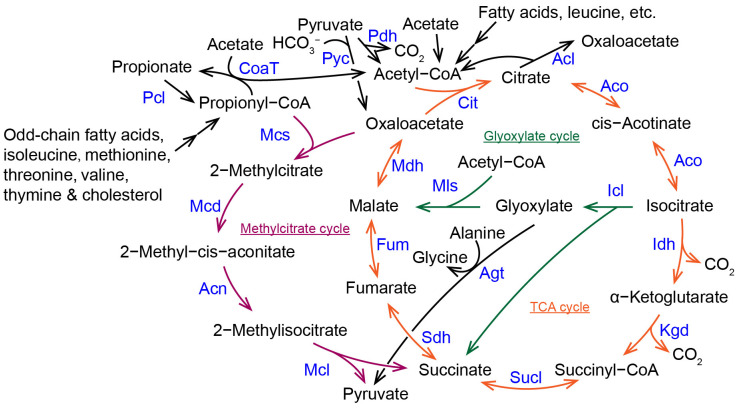
The methylcitrate cycle and its linkage with the TCA and glyoxylate cycles in fungi. Acl, ATP citrate lyase; Acn, aconitase; Aco, aconitase; Agt, alanine, glyoxylate aminotransferase; Cit, citrate synthase; CoaT, CoA-transferase; Fum, fumarase; Icl, isocitrate lyase; Idh, isocitrate dehydrogenase; Kgd, α-ketoglutarate dehydrogenase; Mcd, 2-methylcitrate dehydratase; Mcl, 2-methylisocitrate lyase; Mcs, 2-methylcitrate synthase; Mls, malate synthase; Mdh, malate dehydrogenase; Pcl, propionate-CoA ligase; Pdh, pyruvate dehydrogenase; Pyc, Pyruvate carboxylase; Sdh, succinate dehydrogenase; Sucl, Succinyl-CoA ligase (succinyl-CoA synthetase).

Fungi metabolize acetyl-CoA through the methylcitrate cycle. The methylcitrate cycle shares several metabolic steps with the tricarboxylic acid (TCA) cycle and the glyoxylate pathway (Figure 1). The TCA cycle is the central pathway of carbon metabolism in all organisms. The glyoxylate cycle is the link between lipid and ketogenic amino acid catabolism and gluconeogenesis pathways in fungi and plants. In the methylcitrate cycle, 2-methylcitrate synthase (Mcs) catalyzes propionyl-CoA and oxaloacetate to produce 2-methylcitrate. Then, 2-methylcitrate is converted to 2-methyl-cis-aconitate and 2-methylisocitrate sequentially by 2-methylcitrate dehydratase (Mcd) and aconitase (Acn). Next, 2-methylisocitrate lyase (Mcl) catalyzes 2-methylisocitrate to cleave into pyruvate and succinate [7]. Pyruvate and succinate then enter the TCA cycle, gluconeogenesis and other metabolic pathways [10]. In some bacteria, propionyl-CoA is also metabolized by the methylcitrate cycle [11].

## 2. Function of Methylcitrate Cycle in Pathogenic Fungi

### 2.1. Carbon and Nitrogen Source Utilization and Mycelial Growth

The methylcitrate cycle is an important pathway of carbon metabolism in organisms. Interruption of the methylcitrate cycle leads to the accumulation of intermediate metabolites such as propionyl-CoA in 2-methylcitrate synthase-deficient mutants, 2-methylcitrate in 2-methylcitrate dehydratase-deficient mutants, and 2-methylisocitrate in 2-methylisocitrate lyase-deficient mutants, which are cytotoxic to cells. Excessive accumulation of these products will inhibit the activity of various dehydrogenases in cells, thereby inhibiting cell growth [1,12]. The growth of the methylcitrate cycle-deficient mutants is severely inhibited in carbon and nitrogen sources that are metabolized to produce propionyl-CoA directly. Knock-out mutants of the gene encoding 2-methylcitrate synthase, such as Δ*Momcs1* of *Magnaporthe oryzae* [13], Δ*AfmcsA* of *Aspergillus fumigatus* [14], and Δ*AnmcsA* of *Aspergillus nidulans* [10], failed to grow on media with propionate as the sole carbon source. A 2-methylcitrate dehydratase encoding gene deletion mutant, Δ*Tmmcd* of *Talaromyces marneffei* [15], and knock-out mutants of 2-methylisocitrate lyase encoding genes, including Δ*Momcl1* of *M. oryzae* [13], Δ*Gzmcl1* of *Gibberella zeae* [16], Δ*AnmclA* of *A. nidulans* [17], and Δ*Tamcl* of *Trichoderma atroviride* (a biocontrol fungus) [18], were also unable to grow on media with propionate as the sole carbon source. Valerate, isoleucine, threonine, valine, methionine, or cholesterol are metabolized to produce propionyl-CoA. Δ*Tmmcd* grew slowly on media using valerate, valine, methionine, isoleucine, or cholesterol as the carbon source [15]. Δ*AfmcsA* colony growth was inhibited when valine, isoleucine, or methionine was used as the nitrogen source [14]. Δ*Momcl1* did not grow or grew very slowly on media using threonine, isoleucine, valine or methionine as the sole amino acids [13]. Growth of Δ*Momcs1* was also slowed on media using isoleucine, valine, or methionine as the sole nitrogen source [13]. In glycerol, glucose, or acetate media, the addition of propionate inhibited the growth of Δ*AnmcsA*, Δ*AnmclA*, and Δ*AfmcsA* more severely than the wild type [10,14,17].

Growth of most methylcitrate cycle-deficient mutants is also inhibited in media using carbon and nitrogen sources that did not directly produce propionyl-CoA. Mutants Δ*Momcs1*, Δ*Momcl1*, Δ*Tamcl*, and Δ*Gzmcl1* grew slowly when glucose was used as the carbon source [13,16,18]. Δ*Momcl1* grew slowly on media using glutamic acid (not producing propionyl-CoA) or inorganic nitrogen NaNO_3_ as a nitrogen source [13]. Δ*Tamcl* also grew slowly on PDA medium or media with acetate and ethanol (C2), pyruvate (C3), butyrate (C4), citrate (C6), Tween 20 (C58), N-acetylglucosamine (NAG), or chitin as the sole carbon source [18]. However, Δ*Gzmcl1* grew normally in acetate, Tween 60, and linoleic acid media [16]. Δ*Momcl1* also grew normally when olive oil was the sole carbon source [13]. This is because the glucose metabolism, lipid metabolism, amino acid metabolism and nucleotide metabolism in cells will normally produce endogenous propionyl-CoA. However, the phenotypes of the methylcitrate cycle-deficient mutants in different fungal strains are diverse, which is related to the different types and amounts of intracellular accumulated intermediates.

Within the same fungal strain, the phenotypes caused by the deletion of different genes of the methylcitrate cycle are diverse, which is also related to the type and quantity of the intermediate compounds accumulated in the mutants. The functions of two methylcitrate cycle genes (*MoMCS1* and *MoMCL1*) had been studied in *M. oryzae* [13]. The growth of Δ*Momcs1* on the media using propionyl-CoA-producing amino acids (isoleucine, valine, and methionine) as the sole amino acids was reduced, but to a lesser extent than Δ*Momcl1* [13]. When culturing on the medium with glutamic acid or inorganic nitrogen NaNO_3_ as a sole nitrogen source (not to produce propionyl-CoA directly), the growth of Δ*Momcs1* was normal, while the growth of Δ*Momcl1* was blocked [13]. Δ*Momcs1* grew normally in minimal medium (MM) with glucose as the carbon and energy source, but grew slower in complete medium (CM) containing glucose and peptone. The growth of Δ*Momcl1* was slowed in both MM and CM media. The addition of 0.002% propionate to the MM medium further inhibited the growth of Δ*Momcl1* but not Δ*Momcs1*. The growth of Δ*Momcs1*Δ*Momcl1* in the MM medium and MM medium supplemented with 0.002% propionate was similar to Δ*Momcs1* but different from Δ*Momcl1*. This difference in the growth phenotype of Δ*Momcs1* and Δ*Momcl1* in different carbon and nitrogen sources is related to the different intermediate metabolites accumulated in fungal cells: propionyl-CoA was accumulated in Δ*Momcs1* and Δ*Momcs1*Δ*Momcl1* cells, while 2-methylisocitrate was accumulated in Δ*Momcl1* [13].

### 2.2. Pathogenicity

In animal and plant pathogenic fungi, the methylcitrate cycle is required for pathogenic fungal virulence. However, knockout mutants of different genes in the pathway have different phenotypes, which are related to the type of intermediate compounds accumulated in the mutants. In *M. oryzae*, knocking out *MoMCL1* resulted in a significant reduction in the virulence on plants, while the virulence of Δ*Momcs1* was normal [13]. In *G. zeae*, the virulence of Δ*Gzmcl1* to barley was weakened, while its virulence to wheat was normal [16]. In *T. marneffei*, a pathogen of fatal systemic fungal diseases, Δ*mcd* (deletion of *MCD*, a gene encoding a 2-methylcitrate dehydratase) showed an attenuated virulence in mice [15]. *A. fumigatus*, which causes Aspergillosis in animals and humans, utilizes amino acids from the host as a source of nutrition. The 2-methylcitrate synthase (McsA) is essential for the invasive Aspergillosis, and Δ*AfmcsA* have reduced virulence [14,19]. Moreover, the addition of sodium propionate to the culture medium killed the Δ*AfmcsA* mutant [19]. In a pathogenic fungus *Paracoccidioides lutzii*, which causes Paracoccidioomycosis (PCM), a chemical compound (ZINC08964784) inhibits fungal growth by binding to the 2-methylcitrate synthase [20]. *Trichoderma atroviride,* a kind of biological control fungi, can control the harm of *Botrytis cinerea* and other pathogenic fungi. The inhibitory effect of Δ*Taicl2* (=Δ*Tamcl1*) on the growth of *B. cinerea* was decreased [18].

### 2.3. Asexual and Sexual Reproduction

The methylcitrate cycle affects the asexual reproduction process of fungi. In *M. oryzae*, the ability of Δ*Momcs1* and Δ*Momcl1* to produce spores was significantly reduced [13]. In *A. nidulan*, the addition of 20 mM propionate almost made Δ*AnmclA* unable to produce spores [17]. With regard to fungal sexual reproduction, the ability of Δ*Gzmcl1* to form perithecia is not affected in *G. zeae* [16].

### 2.4. Toxins and Melanin Synthesis

In *A. nidulans* and *A. fumigatus*, Δ*AnmcsA* and Δ*AfmcsA* produce fewer polyketide toxin (such as carcinogens, mycotoxins, and sterigmatocystin) and conidiospore pigment [14,21]. Propionyl-CoA-producing carbon or nitrogen sources such as propionate, heptadecanoic acid, isoleucine, and methionine inhibits polyketide and conidiospore pigment synthesis of *A. nidulans* [22]. The spore pigment synthesis of Δ*AnmcsA* was blocked, and the color of the mutant’s conidia changed from green or yellow to white [10]. Adding exogenous propionate in the medium aggravated this mutant phenotype. In Δ*AnmcsA*, excessive accumulation of acetyl-CoA inhibits the activity of polyketide synthase [21]. Knockout of *PCSA*—a gene encoding a putative propionyl-CoA synthase—in Δ*AnmcsA* reduced the amount of intracellular propionyl-CoA and allowed the mutant to regain the ability to synthesize polyketides [21].

### 2.5. Other Physiological Processes

In *M. oryzae*, the ratio of NAD^+^/NADH in the Δ*Momcs1* aerial mycelium decreased, and the content of nitric oxide (NO) also decreased, meaning that the methylcitrate cycle is involved in the cellular redox state and NO signaling [13]. The altered NAD^+^/NADH ratio may be related to the inhibition by propionyl-CoA of enzymatic activities of metabolic pathways such as the TCA cycle [23]. In yeast *S. cerevisiae*, propionic acid promotes endocytosis, and disrupts cell cycle and cellular respiration [24].

## 3. Relationship between Citrate Synthase of the TCA Cycle and 2-Methylcitrate Synthase in the Methylcitrate Cycle

The TCA cycle is a ubiquitous metabolic pathway in aerobic organisms. It is the final metabolic pathway for the three nutrients (carbohydrates, lipids, and amino acids), and is the hub of the metabolic linkage of carbohydrates, lipids, and amino acids. In eukaryotes, The TCA cycle acts in the mitochondria and is closely related to the respiratory chain. Nutrients are catabolized in cells to produce acetyl-CoA, which is condensed with oxaloacetate to produce citric acid by citrate synthase (Cit or Cs), and then repeatedly dehydrogenated and decarboxylated to produce H_2_O, CO_2_, and reduction equivalents by complete oxidation and decomposition, and to finally re-produce oxaloacetate to enter the next cycle (Figure 1).

**Figure 2:**
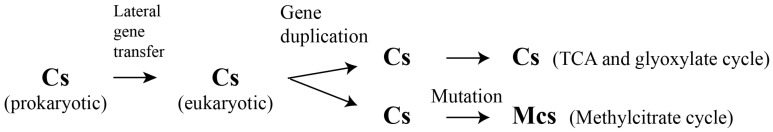
Proposed scheme of the evolution of fungal 2-methylcitrate synthases. The sequences of the fungal 2-methylcitrate synthases (Mcs) are much more homologous to the fungal and bacterial citrate synthases (Cs or Cit) than to the bacterial 2-methylcitrate synthase (PrpC).

The 2-methylcitrate synthase in the methylcitrate cycle shares a common origin with the citrate synthase in the TCA cycle [25] (Figure 2). *S. cerevisiae* has three homologous citrate synthases. Among them, Cit1 is a mitochondrial-specific citrate synthase, Cit2 is a peroxisomal citrate synthase and 2-methylcitrate synthase, and Cit3 is a mitochondrial citrate synthase and 2-methylcitrate synthase [26,27]. In peroxisomes, yeast Cit2 participates in the glyoxylate cycle while condensing propionyl-CoA and oxaloacetate to produce 2-methylcitrate [26]. In yeast *Yarrowia lipolytica*, Cit1 is a bifunctional enzyme: citrate synthase and 2-methylcitrate synthase, while Cit2 is a specific citrate synthase [28]. In bacteria (such as *Escherichia coli*, *Eubacterium* DS2-3R, *Thermoplasma acidophilum*, and *Pyrococcus furiosus*), in addition to GltA (a citrate synthase), PrpC (a 2-methylcitrate synthase) also has partial citrate synthase activity [29].

Schlachter et al. reported the protein crystal structures of an *A. fumigatus* 2-methylcitrate synthase (McsA) and a human citrate synthase (hCS) [25]. The two enzymes have similar structural features and significant sequence homology, but McsA and hCS show significant differences in substrate specificity and cooperativity. hCS and McsA both form a homodimer containing two active sites. The active sites of McsA and hCS are similar. In an active site, McsA contains two histidines (His269 and His351 from chain A) and three arginines (Arg360 and Arg434 from chain A, and Arg454 from chain B), whereas hCS also has two histidines (His265 and His347 from chain A) and three arginines (Arg356 and Arg428 from chain A, and Arg448 from chain B); these amino acid residues are bound to oxaloacetate. The binding of CoA to hCS and McsA appears to be similar except for the difference in an amino acid residue of chain B: in hCS, Arg73 of chain A binds to P2 of CoA and Arg191 of the B chain binds to ribose of CoA; in McsA, however, Arg74 chain A binds to P2 of CoA, and Lys192 of chain B forms a single salt bridge with the ribose sugar of CoA. The only difference observed near the CoA binding site is the presence of Ala348 for hCS and Gly352 for McsA. The G352A mutation in McsA does not have a significant effect on substrate binding and conformational changes, but the A348G mutation in hCS is much more obvious. Considering that the active sites of both enzymes are almost identical, differences in the amino acid residues near the active sites cause differences in the reactions catalyzed by the enzymes [25]. In addition, hCS has no 2-methylcitrate synthase activity, whereas McsA has a citrate synthase activity [19].

## 4. Relationship between Isocitrate Lyase of the Glyoxylate Cycle and 2-Methylisocitrate Lyase of the Methylcitrate Cycle

Within peroxisomes, the glyoxylate cycle converts 2-carbon acetyl-CoA into 4-carbon succinate, which can be utilized for de novo gluconeogenesis. The two key enzymes in this cycle are malate synthase (Mls) and isocitrate lyase (Icl). Malate synthase condenses the first acetyl-CoA with glyoxylate to form malate, which is oxidized to oxaloacetate. Then, citrate synthase (Cit) condenses the second acetyl-CoA with oxaloacetate to form citrate; citrate is then converted to isocitrate. Finally, isocitrate lyase cleaves isocitrate to regenerate glyoxalate as well as 4-carbon succinate. Except for malate synthase and isocitrate lyase, the rest of the enzymes of the glyoxylate cycle are the same as the TCA cycle (Figure 1). The glyoxylate cycle allows pathogenic fungi to utilize lipid, ethanol, and acetate as the sole carbon sources and is necessary for fungal growth, development, and virulence [30,31].

**Figure 3:**
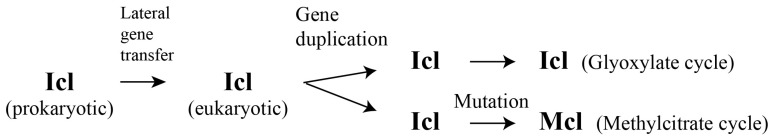
Proposed scheme of the evolution of fungal 2-methylisocitrate lyases. Fungal 2-methylisocitrate lyases (Mcl) show much higher homology to the fungal and bacterial isocitrate lyases (Icl) than to the bacterial 2-methylisocitrate lyase (PrpB).

The 2-methylisocitrate lyase of the methylcitrate cycle and isocitrate lyase of the glyoxylate cycle share a common origin [32] (Figure 3). Bacteria have isocitrate lyase (Icl) and 2-methylisocitrate lyase (PrpB). However, bacterial 2-methylisocitrate lyase is very distantly related to fungal 2-methylisocitrate lyase. The wild-type isocitrate lyase of *E. coli* and *A. fumigatus* has both isocitrate lyase and 2-methylisocitrate lyase activities. Due to its very high Km value to bind 2-methylisocitrate (Km = 213 mM), *A. fumigatus* wild-type isocitrate lyase did not have obvious 2-methylisocitrate lyase activity in vivo [32]. Based on phylogenetic analysis and experimental validation, Müller et al. proposed that the fungal 2-methylisocitrate lyase evolved from fungal isocitrate lyase by gene duplication, and fungal isocitrate lyase was acquired from earlier eukaryotes from prokaryotes via horizontal gene transfer [32]. 

Comparison of *A. fumigatus* isocitrate lyase and 2-methylisocitrate lyase showed a 45% sequence identity. The conserved phenylalanine 455 and threonine 457 in isocitrate lyase were mutated to the conserved leucine (F455L) and serine (T457S) in 2-methylisocitrate lyase, respectively [32]. Single mutations or double mutations in both amino acids of the isocitrate lyase strongly increased the 2-methylisocitrate lyase activity and decreased the isocitrate lyase activity. Among them, the F455L mutation had a stronger effect on isocitrate lyase activity than the T457S mutation. Meanwhile, the *E. coli* isocitrate lyase double mutant (F349L/T351P) had a catalytic efficiency and characteristics of 2-methylisocitrate lyase very similar to that of the *A. fumigatus* isocitrate lyase double mutant (F455L/T457S). This suggests that F455L represents the key mutation for loss of isocitrate lyase function, whereas T457S is the key mutation for gaining 2-methylisocitrate lyase function. Mutations in both active-site residues convert an isocitrate lyase, whether it is of bacterial or fungal origin, into a 2-methylisocitrate lyase [32].

However, after the conserved leucine and serine residues in *A. fumigatus* 2-methylisocitrate lyase were mutated to the conserved phenylalanine and threonine residues in isocitrate lyase (L521F and S523T), the mutant enzyme displays the catalytic properties of 2-methylisocitrate lyase that are highly similar to those of the wild-type enzyme [33]. The mutant enzyme has increased its affinity for isocitrate binding, but only shows slight isocitrate lyase activity. Further mutations at additional sites of the 2-methylisocitrate lyase seem to prevent the turnover of the bound substrate [32]. 

In *S. cerevisiae*, Icl2 is a specialized 2-methylisocitrate lyase, whereas Icl1 is an isocitrate lyase with partial 2-methylisocitrate lyase activity. In *G. zeae*, a single knockout of *GzICL1* caused a growth defect on the sodium acetate medium, whereas a knockout of *GzMCL1* caused a defect in the utilization of sodium propionate. In *M. oryzae*, knockout of *MoMCL1* caused defective utilization of sodium propionate [13], whereas knockout of *MoICL1* caused defective utilization of lipid and sodium acetate [31]. Because of the high degree of protein sequence identity between 2-methylisocitrate lyase and isocitrate lyase, an isocitrate lyase is named as Icl1, whereas a 2-methylisocitrate lyase is referred to Icl2 in some fungal species. This nomenclature caused confusion in correctly and conveniently distinguishing these two enzymes, such as in *M. oryzae*, where Icl1 and Icl2 are sometimes misinterpreted to isocitrate lyases [34,35]. Therefore, it is suggested that Mcl1 but not Icl2 is used to refer to a 2-methylisocitrate lyase and Icl1 to a citrate lyase.

## 5. Compartmentalization of the TCA Cycle, the Methylcitrate Cycle, and the Glyoxylate Cycle In Vivo

There are overlapping metabolic steps and enzymes between the methylcitrate cycle, the glyoxylate cycle, and the TCA cycle (Figure 1), and despite differences in substrate and catalytic characteristics, some enzymes originate from the same ancestral proteins [25,32]. Enzymes acting in different metabolic cycles correlate with temporal and spatial expression, subcellular localization, and substrate specificity. Compartmentalization of metabolic activities allows individual physiological activities within a cell to be segregated from each other and performed sequentially in an orderly manner, increasing the efficiency of the activities. The TCA cycle occurs within the mitochondria of eukaryotes, but it has recently been found that the TCA cycle is also present in the nuclei of mammalian and plant cells [36,37,38,39]. The methylcitrate cycle is split between mitochondria and cytoplasm [40]. The glyoxylate cycle is segregated in peroxisomes and cytoplasm [41]. These three cycles occur in distinct and overlapping organelles, adding to the complexity of cellular carbon metabolism processes.

**Figure 4:**
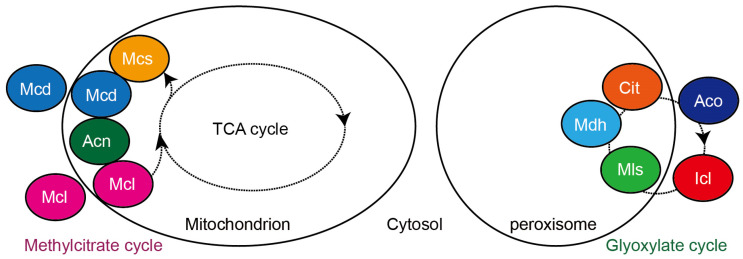
Localization of fungal proteins involved in the methylcitrate cycle and glyoxylate cycle in mitochondria, peroxisomes, and cytoplasm in fungi. Acn, aconitase or 2-methylisocitrate dehydratase; Aco, aconitase; Cit, citrate synthase; Icl, isocitrate lyase; Mcd, 2-methylcitrate dehydratase; Mcl, 2-methylisocitrate lyase; Mcs, 2-methylcitrate synthase; Mls, malate synthase; Mdh, malate dehydrogenase.

The enzymes of the methylcitrate cycle are located in the mitochondria and cytoplasm (Figure 4). 2-methylisocitrate lyase (Icl2) and citrate synthase 3 (Cit3) in *S. cerevisiae* [42,43], 2-methylisocitrate lyase (Mcl1) in *M. oryzae* [13], 2-methylcitrate synthase in *A. fumigatus* [14], three enzymes (2-methylcitrate synthase, 2-methylcitrate dehydratase, and 2-methylisocitrate dehydratase) in *Y. lipolytica* [8], and 2-methylcitrate synthase in *Toxoplasma gondii* (a one-celled eukaryotic parasite) [40] are localized in the mitochondria. However, 2-methylcitrate dehydratase is in the cytoplasm, and 2-methylisocitrate lyase is in the cytoplasm surrounding the mitochondria in *T. gondii* [40]. 2-methylisocitrate lyase in *Y. lipolytica* is located in both the mitochondria and cytoplasm [8]. Interestingly, yeast Cit2 is a peroxisomal citrate synthase, but can act as a 2-methylcitrate synthase condensing propionyl-CoA and oxaloacetate within peroxisomes to produce 2-methylcitrate [26].

The glyoxylate cycle is split into two parts, one in the peroxisome and another in the cytoplasm (Figure 4). The enzymes of the glyoxylate cycle are located in the mitochondria and cytoplasm. Kunze et al. reviewed the relationship between the function of the glyoxylate cycle and the distribution of the individual enzymes in the peroxisomes and the cytoplasm [41]. In the human fungal pathogen *C. albicans*, isocitrate lyase (Icl1) and malate synthase (Mls1) are localized to peroxisomes. This peroxisomal localization is dependent on the PTS1 receptor Pex5p [44]. In the Δ*Capex5* mutant, Icl1 and Mls1 were localized to the cytosol but could grow normally in acetate and ethanol media. The Δ*Cafox2* mutant that completely lacked fatty acid β-oxidation but had no peroxisomal protein input defects showed significantly reduced growth on nonfermentable carbon sources such as acetate and ethanol. When Icl1 and Mls1 were relocated to the cytoplasm by deletion of the *PEX5* gene, Δ*Cafox2*Δ*Capex5* restored the growth of the Δ*Cafox2* mutant on these carbon compounds [44]. In the mycorrhizal ascomycete fungus *Tuber borchii*, immunofluorescence co-localization showed that isocitrate lyase (TbIcl) co-localized with the peroxisomal marker protein Fox2, and was distributed in the peroxisome [45]. *S. cerevisiae* Cit2 is involved in the glyoxylate cycle of the peroxisome, whereas Cit1 and Cit3 are involved in the mitochondrial TCA cycle and the methylcitrate cycle [26,27]. *S. cerevisiae* has three malate dehydrogenases: Mdh1 is localized in the mitochondria and participates in the TCA cycle, whereas Mdh3 is localized in the peroxisome and participates in the glyoxylate cycle [30,46]. Mdh2 is located in the cytoplasm and participates in gluconeogenesis [47]. However, Mdh2 is also piggybacked into the peroxisome via association with Mdh3 and a Pex5-dependent piggybacking mechanism and participates in the glyoxylate cycle [48].

## 6. Interplay between the TCA Cycle, the Methylcitrate Cycle, and the Glyoxylate Cycle

**Figure 5:**
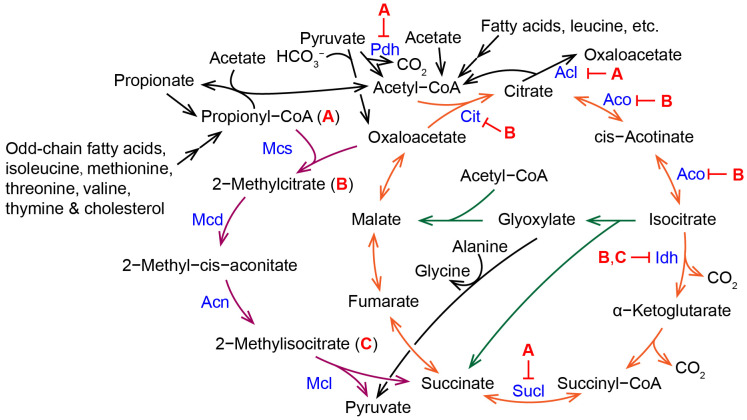
The intermediates propionyl-CoA (**A**), 2-methylcitrate (**B**), and 2-methylisocitate (**C**) from the methylcitrate cycle inhibit the activity of enzymes of the TCA cycle. Acl, ATP citrate lyase; Acn, aconitase; Aco, aconitase; Cit, citrate synthase; Idh, isocitrate dehydrogenase; Mcd, 2-methylcitrate dehydratase; Mcl, 2-methylisocitrate lyase; Mcs, 2-methylcitrate synthase; Pdh, pyruvate dehydrogenase; Sucl, Succinyl-CoA ligase (succinyl-CoA synthetase).

Propionate inhibited the growth of the fungus *A. nidulans* on the glucose medium, but not on the acetate medium [23]. Δ*AnmcsA* is more sensitive to sodium propionate than the wild type and accumulates 10-fold more propionyl coenzyme A in vivo [23]. Inhibition of fungal growth by propionate is associated with its high accumulation of intermediate metabolites in the methylcitrate cycle, such as propionyl coenzyme A, 2-methylcitrate, and 2-methylioscitrate [13] (Figure 5). In *A. nidulans*, propionyl-CoA inhibits the activity of CoA-dependent enzymes such as pyruvate dehydrogenase, succinyl-CoA synthetase, and ATP citrate lyase [23]. Accumulation of 2-methylcitrate in the Δ*mcd* mutant and 2-methylioscitrate in the Δ*mcl* mutant also severely inhibits enzyme activity and carbon metabolism. For example, 2-methylisocitrate inhibits NADP-dependent isocitrate dehydrogenase in *A. nidulans* [17], and 2-methylcitrate affects the TCA cycle by competitively inhibiting citrate synthase, aconitase, nicotinamide adenine dinucleotide^+^ (NAD^+^)- and NADP^+^-linked isocitrate dehydrogenase, phosphofructokinase, and the tricarboxylase carrier in human [12].

Addition of acetate eliminated the inhibitory effect of propionate on fungal growth of the wild-type as well as the growth and developmental defects of the methylcitrate cycle-deficient mutants, which was associated with an increase in the amounts of accumulated intermediates of the methylcitrate cycle in cells [13,23,49]. In *A. nidulans*, the addition of acetate, but not ethanol, to glucose/propionate medium reduced intracellular levels of propionyl-CoA [23,49]. This result is related to the function of a class III CoA-transferase CoaT (Figure 1). CoaT is localized in the mitochondria. CoaT uses only acetyl-CoA, propionyl-CoA, succinyl-CoA, and their corresponding free acids as donors and acceptors, respectively. The substrate couple succinyl-CoA and acetate had the highest specific activity, followed by succinyl-CoA and propionate, and propionyl-CoA and acetate. The Δ*AncoaT* mutant grew normally under conditions in which glucose, acetate, glycerol, or ethanol was the sole carbon source. However, Δ*AncoaT* was more sensitive to the addition of propionate, especially when glycerol was used as a second carbon source. The wild-type, Δ*AncoaT*, and Δ*AnmcaA* could sporulate in media supplemented with acetate and propionate, whereas Δ*AncoaT*Δ*AnmcaA* could not sporulate in media supplemented with acetate and propionate. Therefore, it is believed that CoaT detoxifies the cells by transferring the CoASH moiety from propionyl-CoA to acetate, reducing propionyl-CoA as well as other intermediate metabolites in the cells [49]. A class III CoA-transferase CoaT gene (*MGG_06609*) is also present in the *M. oryzae* genome. The addition of acetate restored spore production of Δ*Momcs1* and Δ*momcl1* of *M. oryzae* in the complete medium [13]. Addition of acetate eliminated the growth inhibition of propionate on the wild-type and Δ*momcs1*, but the effect on Δ*momcl1* was relatively poor, which was related to the accumulation of not only propionyl-CoA but also 2-methylcitrate and 2-methylisocitrate in Δ*momcl1* [13].

In *M. oryzae*, the virulence of both Δ*momcl1* and Δ*moicl1* was reduced, while the decrease in virulence of Δ*momcl1*Δ*moicl1* (in which both the methylcitrate cycle and the glyoxylate cycle were disrupted) was even more significant [13]. In *G. zeae*, both Δ*Gzmcl1* and Δ*gzicl1* had normal virulence on wheat, but Δ*Gzmcl1*Δ*gzicl1* displayed significantly reduced virulence [16]. The growth of Δ*momcl1*Δ*moicl1* and Δ*momcl1* was slowed in the glucose medium, whereas the growth of Δ*Momcl1*Δ*Moicl1* and Δ*Moicl1* were slowed in the olive oil medium [13]. These facts suggest a synergistic relationship between the methylcitrate cycle and the glyoxylate cycle in carbon metabolism and virulence. In *M. oryzae*, the expression of *MoMCL1* and *MoMCS1* was increased not only in propionyl-CoA-producing carbon sources, but also in acetyl-CoA-producing carbon sources, further suggesting that the methylcitrate cycle is closely metabolically linked to lipolysis, the glyoxylate cycle, and the TCA cycle [13]. In propionic aciduria patients (human), accumulation of propionyl-CoA and 2-methylcitrate leads to abnormal mitochondrial function [1,12]. Abnormal mitochondrial function leads to abnormalities in other carbon metabolic processes such as the TCA cycle and β-oxidation. In the pathogenic fungus *Paracoccidioides* spp., propionyl-CoA inhibits the expression of *SUCLA* (encoding a succinyl-CoA ligase) and *PDH* (encoding a pyruvate dehydrogenase) of the TCA cycle, and remodels the fungal metabolic pathways [50]. The addition of 2-methylcitrate synthase inhibitors also altered the carbon metabolic pathways of *Paracoccidioides brasiliensis* [51]. In *Y. lipolytica*, the Δ*Ylphd1* mutant (inactivation of the 2-methylcitrate dehydratase) has an increased utilization of glycerol [52]. In the nitrogen-limited medium with glycerol as substrate, Δ*Ylphd1* altered intracellular carbon metabolism pathways to promote glycerol utilization and increase greater acetate production and lipid accumulation [52]. These data suggest that altering the methylcitrate cycle also affects both the glyoxylate cycle and the TCA cycle.

## 7. The Methylcitrate Cycle as a Potential Target for Antifungal Compounds

Because blocking the methylcitrate cycle disrupts the function of the TCA cycle and mitochondria, thereby interfering with the metabolic activity and growth of pathogenic fungi, methylcitrate cycle enzymes can be used as targets for antifungal drugs. For example, Lima et al. screened six compounds from 89,415 compounds that were able to inhibit the enzymatic activity of recombinant PiMcs in vitro. Among them, only one compound, ZINC08964784, inhibited the biological activity of *Paracoccidioides* spp. cells [20]. Further analysis revealed that the fungal cells undergo a metabolic shift when exposed to ZINC08964784: glycolysis and TCA cycle were down-regulated, while β-oxidation was upregulated, proteolytic enzyme expression was increased, amino acids were degraded for energy production, and reactive oxygen species levels were increased [51]. ZINC08964784 is non-cytotoxic to mammalian cells and has a very high selectivity index. Therefore, ZINC08964784 has therapeutic potential for the treatment of Paracoccidioomycosis [20,51].

## 8. Regulation of the Methylcitrate Cycle and the Glyoxylate Cycle

The fungal methylcitrate cycle and glyoxylate cycle are regulated by carbon catabolite repression and derepression (CCR and CCDR). Propionate promotes the expression of genes involved in the methylcitrate cycle [13,16,50]. In *S. cerevisiae*, the expression of *ICL2* (encoding 2-methylisocitrate lyase) and *ICL1* (encoding isocitrate lyase with a low 2-methylisocitrate lyase activity) is repressed by glucose and induced by ethanol or threonine [41,53]. In *M. oryzae*, the expression of *MoICL1* and *MoMCL1* was repressed by glucose and induced by lipid [54]. When glucose is used as the carbon source, the phosphatase Smek1 activates the carbon catabolite repressor CreA and inhibits the expression of *MoICL1* and *MoMCL1*; while lipid is used as the carbon source, Smek1 activates the transcription activator Crf1 and promotes the expression of *MoICL1* and *MoMCL1* [34,54].

## 9. Future Perspectives

The methylcitrate cycle, glyoxylate cycle, and the TCA cycle have been extensively studied and well understood over the past 100 years. However, there are still many unanswered questions about the evolutionary relationship among the three carbon cycles, the spatial separation of the three carbon cycles, the transport of intermediary metabolites between different organelles, and the differentiation of the functions of related enzymes between the three carbon cycles. Propionyl-CoA is produced by metabolic processes such as lipid metabolism, amino acid metabolism, and nucleic acid metabolism. These metabolic processes occur in different organelles, and the resulting propionyl-CoA is distributed in various organelles such as mitochondria and peroxisomes. The enzymes involved in the methylcitrate cycle that degrade propionyl-CoA are mainly located in the mitochondria, with some also located in the cytoplasm. However, the temporal and spatial mechanisms of the mitochondrial and cytoplasmic distribution of individual enzymes in a given fungal species have not been investigated in detail, as well as the significance of the cleavage of the methylcitrate cycle between different organelles. The 2-methylisocitrate dehydratase of *Y. lipolytica* catalyzes the conversion of 2-methyl-cis-aconitate to 2-methylisocitrate [8], but its homologue in pathogenic fungi such as *M. oryzae* is 2-methylcitrate-dehydratase. The sequence of 2-methylisocitrate dehydratase is also homologous to aconitases. Bacterial aconitases catalyze the conversion of 2-methyl-cis-aconitate to 2-methylisocitrate. In the TCA cycle, aconitases also catalyze the conversion of citrate to isocitrate (Figure 1). There are several aconitases in pathogenic fungi (such as three aconitases in *M. oryzae* [13]) and their functions were still unrevealed. The roles of each aconitase in the pathogenic fungi in both the methylcitrate cycle and the TCA cycle need to be identified in the future. In addition, fungal isocitrate lyase and 2-methylisocitrate lyase share a common origin, but their protein crystal structures have not been resolved. Further resolution of the crystal structures of fungal isocitrate lyase and 2-methylisocitrate lyase will be instrumental in understanding the mechanisms of protein evolution, as well as enzyme-substrate binding and catalytic mechanisms. 

The methylcitrate cycle is critical in the development and infection of pathogenic fungi and is potentially important in biomedical, agricultural, and biotechnological research. A further study to understand the initiation and control mechanism of the methylcitrate cycle and its relationship with the tricarboxylic acid cycle, the glyoxylate cycle, and other metabolisms for lipids, carbohydrates, and amino acids could lead to antifungal products of the devastating fungal diseases worldwide.

## Data Availability

Not applicable.

## Sample Availability

Samples of the compounds are not available from the authors.
